# Supplementary material for: Isolation, Structural Analysis and Biological Activity Assays of Biselisabethoxanes A and B: Two Dissymmetric Bis-Diterpenes from the Southwestern Caribbean Sea Gorgonian Coral Pseudopterogorgia elisabethae
Source: Molecules. 2022 Nov 15;27(22):7879. doi: 10.3390/molecules27227879 (PMC9694721; doi:10.3390/molecules27227879)
Supplement: Supplementary file 1 [file molecules-27-07879-s001.zip › molecules-2012874-supplementary.pdf]

SUPPLEMENTARY MATERIALS

Isolation, Structural Analysis and Biological Activity Assays of Biselisabethoxanes A and B: Two Dissymmetric *Bis*-Diterpenes from the Southwestern Caribbean Sea Gorgonian Coral *Pseudopterogorgia elisabethae*

Ileana I. Rodríguez,[a] Abimael D. Rodríguez,[ab]* and Charles L. Barnes[c]

[a] *Department of Chemistry, University of Puerto Rico, PO Box 23346, UPR Station, San Juan, Puerto Rico 00931-3346, USA*

[b] *Molecular Sciences Research Center, University of Puerto Rico, 1390 Ponce de León Avenue, San Juan, Puerto Rico 00926, USA*

[c] *Department of Chemistry, University of Missouri-Columbia, Columbia, Missouri 65211*

Corresponding Author

*Tel: (787)-523-5320
E-mail: Abimael.rodriguez1@upr.edu

List of Supplementary Materials

Index **Pages**

Table S1. ^{1}H-^{1}H COSY, HMBC and NOESY (500 MHz, $CDCl_3$) data for biselisabethoxane A (**1**) 3

Table S2. ^{1}H-^{1}H COSY, HMBC and NOESY (500 MHz, $CDCl_3$) data for biselisabethoxane B (**2**) 4

Table S3. Charge distribution of ortho-benzoquinone **7** 5

Table S4. Molecular orbital energies for reactants **7** and **7a** 7

Table S5. Crystal and structure refinement data for **1** 8

Figure S1. Predicted structures from self-same dimerization of *ortho*-benzoquinone **7** 9

Figure S2. ^{1}H NMR (500 MHz, $CDCl_3$) spectrum of **1** 10

Figure S3. ^{1}H NMR (500 MHz, $CDCl_3$) spectrum of **1** (*expansion*) 11

Figure S4. ^{13}C NMR (125 MHz, $CDCl_3$) spectrum of **1** 12

Figure S5. ^{13}C NMR (125 MHz, $CDCl_3$) spectrum of **1** (*expansion*) 13

Figure S6. COSY spectrum of **1** 14

Figure S7. HMQC spectrum of **1** 15

Figure S8. HMBC spectrum of **1** 16

Figure S9. HMBC spectrum of **1** (*expansion*) 17

Figure S10. HMBC spectrum of **1** (*expansion*) 18

Figure S11. NOESY spectrum of **1** 19

Figure S12. IR spectrum of **1** 20

Figure S13. ^{1}H NMR (500 MHz, $CDCl_3$) spectrum of **2** 21

Figure S14. ^{1}H NMR (500 MHz, $CDCl_3$) spectrum of **2** (*expansion*) 22

Figure S15. ^{13}C NMR (125 MHz, $CDCl_3$) spectrum of **2** 23

Figure S16. ^{13}C NMR (125 MHz, $CDCl_3$) spectrum of **2** (*expansion*) 24

Figure S17. COSY spectrum of **2** 25

Figure S18. HMQC spectrum of **2** 26

Figure S19. HMQC spectrum of **2** (*expansion*) 27

Figure S20. HMBC spectrum of **2** 28

Figure S21. HMBC spectrum of **2** (*expansion*) 29

Figure S22. HMBC spectrum of **2** (*expansion*) 30

Figure S23. NOESY spectrum of **2** 31

Figure S24. IR spectrum of **2** 32

Table S1. ^{1}H NMR (500 MHz), ^{13}C NMR (125 MHz), ^{1}H–^{1}H COSY, HMBC, and NOESY spectroscopic data for biselisabethoxane A (**1**) in CDCl$_3$[a]

Atom	δ_H	δ_C	^{1}H–^{1}H COSY	HMBC[c]	NOESY
1	3.60	36.8	H2αβ, H14		Me20
2α	1.22	40.2	H1, H2β, H3	H1, Me18	H4
2β	1.94		H1, H2α		
3	1.21	34.0	H2α, H4, Me18	H5β, Me18	H5β, Me18
4	2.01	44.6	H3, H5α	H2αβ, H3, H6αβ, Me18	H2α
5α	0.95	27.7	H4, H5β, H6αβ		Me18
5β	2.03		H5α, H6β		H3, H7
6α	2.15	32.0	H5α, H6β, H7	H7, Me19	
6β	1.30		H5αβ, H6α, H7		
7	3.20	28.6	H6αβ, Me19	H5β, H6αβ, Me19	H5β, Me19
8		126.2		H7, Me19, C9-OH	
9		147.1		H7, C9-OH	
10		137.2		Me20, C9-OH	
11		129.6		H1, Me20	
12		129.6		H1, H14, Me20	
13		135.9		H1, H5β, H7	
14	4.94	131.4	H1, Me16, Me17	H1, Me16, Me17	Me16
15		128.3		H1, Me16, Me17	
16	1.66	25.4	H14	H14, Me17	H14
17	1.70	17.5	H14	H14, Me16	
18	1.02	20.0	H3	H3	H3, H5α
19	1.25	23.1	H7	H7	H7
20	1.96	15.6			H1
9-OH	5.97				
1'		61.6		H5α', H6', H8α'	
2'		141.1		H3', H4α', Me18', C17'-OH	
3'	3.14	28.7	H4β', Me18'	H5α', Me18'	
4α'	1.46	24.3	H4β', H5αβ'	H3', H5β', H6', Me18'	
4β'	1.78		H3', H4α', H5α'		
5α'	1.55	19.3	H4αβ', H5β'	H3'	
5β'	1.80		H4α', H5α', H6'		
6'	2.35	40.3	H5β', H7'	H8α', Me19'	Me18', Me19'
7'	2.05	41.7	H6', H8αβ', Me19'	H6', H8β', Me19'	
8α'	2.22	44.5	H7', H8β'	Me19'	
8β'	0.97		H7', H8α'		
9'	—[b]	53.4[b]		H8αβ'	
10'	—[b]	60.7[b]		Me12', Me13', Me20'	Me19', Me20'
11'		~85.0[b]		Me12', Me13'	
12'	1.01	26.8[b]		Me13'	Me13'
13'	1.02	26.9[b]		Me12'	Me12'
14'		203.1		H6', H9', Me20'	
15'		69.2[b]		Me20'	
16'		~196.0[b]		Me20'	
17'		146.9		H3', C17'-OH	
18'	1.16	17.7	H3'		H6'
19'	1.06	18.1	H7'	H6'	H6', H10'
20'	1.66	15.6			H10'
17'-OH	6.18				

[a] Spectra were recorded at 25°C. Chemical shift values are in ppm relative to TMS (0.00 ppm) or CDCl$_3$ (77.0 ppm) signals. [b] Detection of this signal was complicated by the increased linewidths associated with slower tumbling, and the spectral overlap from the large number of unique signals. Either this signal was not detected, or it appeared as a broad low intensity signal. [c] Protons correlated to carbon resonances in ^{13}C column. Parameters were optimized for $^{2,3}J$ = 6 and 8 Hz.

Table S2. ^1H NMR (500 MHz), ^{13}C NMR (125 MHz), ^1H–^1H COSY, HMBC, and NOESY spectroscopic data for biselisabethoxane B (**2**) in $CDCl_3$[a]

Atom	δ_H	δ_C	^1H–^1H COSY	HMBC[b]	NOESY
1	3.65	36.7	H2αβ, H14		Me20
2α	1.18	40.3	H1, H2β, H3	H1, Me18	H4
2β	1.94		H1, H2α		
3	1.20	34.2	H2α, H4, Me18	H5β, Me18	H5β, Me18
4	1.98	44.4	H3, H5α	H2αβ, H3, H6αβ, Me18	H2α
5α	0.97	28.3	H4, H5β, H6αβ		Me18
5β	2.07		H5α, H6β		H3, H7
6α	2.17	31.8	H5α, H6β, H7	H7, Me19	
6β	1.30		H5αβ, H6α, H7		
7	3.35	27.6	H6αβ, Me19	H5β, H6αβ, Me19	H5β, H14', Me16', Me-17'
8		126.3		H7, Me19, C9-OH	
9		137.2		H7, C9-OH	
10		137.8		Me20, C9-OH	
11		122.3		H1, Me20	
12		129.7		H1, H14, Me20	
13		132.3		H1, H5β, H7	
14	5.00	131.8	H1, Me16, Me17	H1, Me16, Me17	Me16
15		127.6		H1, Me16, Me17	
16	1.63	25.3	H14	H14, Me17	H14
17	1.68	17.5	H14	H14, Me16	
18	1.00	20.0	H3	H3	H3, H5α
19	1.21	23.3	H7	H7	H7, Me20'
20	1.84	11.5			H1
1'		144.7		H2αβ'	
2α'	1.82	42.2	H2β', H3'	Me18'	H2β', Me18'
2β'	2.12		H2α'		H2α', Me18'
3'	1.36	32.8	H2α', H4', Me18'	Me18'	H5αβ', Me18'
4'	1.80	44.2	H3', H5α'	H2β', Me18'	H5β', H6β', Me18'
5α'	1.17	25.3	H4', H5β', H6α'		H3', H7'
5β'	2.00		H5α', H6β'		H4', H6β'
6α'	2.09	31.0	H5α', H6β', H7'	Me19'	H7'
6β'	1.12		H5β', H6α', H7'		H4', H5β'
7'	2.82	29.3	H6αβ', Me19'	Me19'	H5β', H6α', Me19'
8'		129.3		Me19'	
9'		191.2		C10'-OH	
10'		90.1		C10'-OH, Me20'	
11'		78.8		C10'-OH, Me20'	
12'		132.5		H2αβ, H14', Me20'	
13'		157.9		H5β'	
14'	5.71	126.4	Me16', Me17'	Me16', Me17'	H7, Me16', Me20'
15'		129.4		Me16', Me17'	
16'	1.76	25.3	H14'	H14', Me17'	H7, H14'
17'	1.50	19.6	H14'	H14', Me16'	H7
18'	0.95	19.1	H3'		H2αβ', H3', H4'
19'	1.22	22.0	H7'		H7'
20'	1.45	21.0			10'-OH, H14', Me19
10'-OH	4.70				Me20'

[a] Spectra were recorded at 25°C. Chemical shift values are in ppm relative to TMS (0.00 ppm) or $CDCl_3$ (77.0 ppm) signals. [b] Protons correlated to carbon resonances in ^{13}C column. Parameters were optimized for $^{2,3}J = 6$ and 8 Hz.

Table S3. Charge distributions of *ortho*-benzoquinone 7.

Job type: Equilibrium Geometry
Method: ωB97X-D Basis set: 6-31G*
Energy: -927.944232 hartrees

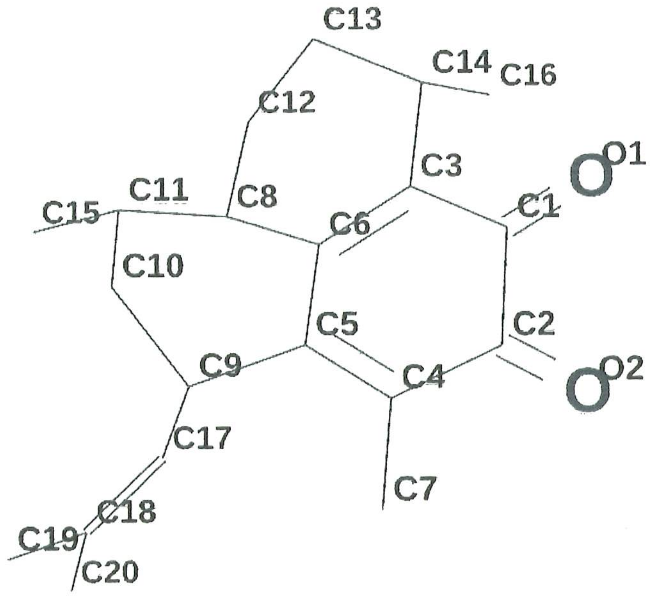

Atom Label	Natural charge	Mulliken charge	Electrostatic charge
C1	+0.490	+0.358	+0.465
C2	+0.486	+0.362	+0.354
C3	-0.102	-0.010	-0.366
C4	-0.096	+0.013	+0.190
C5	+0.035	+0.052	-0.555
C6	+0.024	+0.066	+0.243
C7	-0.728	-0.583	-0.597
C8	-0.284	-0.195	-0.157
C9	-0.310	-0.240	+0.957
C10	-0.456	-0.319	-1.108
C11	-0.254	-0.108	+0.748
C12	-0.464	-0.327	-0.189
C13	-0.466	-0.316	-0.519
C14	-0.284	-0.173	+0.485
C15	-0.691	-0.509	-0.888
C16	-0.688	-0.505	-0.708
C17	-0.247	-0.188	-0.671
C18	+0.000	+0.153	+0.544
C19	-0.719	-0.561	-0.800
C20	-0.708	-0.554	-0.798
H1	+0.252	+0.184	+0.155
H2	+0.250	+0.156	-0.010
H3	+0.251	+0.183	+0.164

H4	+0.273	+0.214	+0.190
H5	+0.240	+0.165	+0.209
H6	+0.266	+0.171	+0.089
H7	+0.240	+0.158	+0.128
H8	+0.246	+0.162	+0.098
H9	+0.271	+0.172	-0.026
H10	+0.244	+0.173	+0.217
H11	+0.250	+0.166	+0.215
H12	+0.244	+0.164	+0.109
H13	+0.250	+0.161	+0.168
H14	+0.239	+0.171	+0.220
H15	+0.278	+0.184	+0.019
H16	+0.238	+0.164	+0.165
H17	+0.235	+0.163	+0.186
H18	+0.250	+0.164	+0.267
H19	+0.227	+0.157	+0.190
H20	+0.256	+0.195	+0.201
H21	+0.231	+0.150	+0.209
H22	+0.240	+0.176	+0.184
H23	+0.249	+0.178	+0.220
H24	+0.250	+0.180	+0.219
H25	+0.245	+0.175	+0.215
H26	+0.244	+0.176	+0.202
O1	-0.500	-0.440	-0.428
O2	-0.496	-0.439	-0.403

Table S4. HOMO-LUMO energies for the heterodiene 7 and heterodienophile 7a.

Reactant	HOMO Energy (eV)	LUMO Energy (eV)
ortho-Quinone 7	–8.23	–1.06
Enol 7a	–7.28	–0.45

Table S5. Crystal and structure refinement data for 1

Table 1. Crystal data and structure refinement

Identification code	Ito-f4126
Empirical formula	C20 H27 O2.50
Formula weight	307.42
Temperature	173(2) K
Wavelength	0.71073 A
Crystal system, space group	Monoclinic, P2~1~
Unit cell dimensions	a = 12.1072(6) A alpha = 90 deg.<br>b = 10.5771(6) A beta = 95.6550(10) deg.<br>c = 27.4452(14) A gamma = 90 deg.
Volume	3497.5(3) A^3
Z, Calculated density	8, 1.168 Mg/m^3
Absorption coefficient	0.075 mm^-1
F(000)	1336
Crystal size	0.50 x 0.25 x 0.05 mm
Theta range for data collection	1.69 to 27.13 deg.
Limiting indices	-14<=h<=15, -13<=k<=13, -35<=l<=28
Reflections collected / unique	22255 / 8141 [R(int) = 0.0757]
Completeness to theta = 27.13	99.4 %
Absorption correction	Semi-empirical from equivalents
Max. and min. transmission	0.98 and 0.85
Refinement method	Full-matrix least-squares on F^2
Data / restraints / parameters	8141 / 1 / 848
Goodness-of-fit on F^2	0.847
Final R indices [I>2sigma(I)]	R1 = 0.0440, wR2 = 0.0689
R indices (all data)	R1 = 0.1356, wR2 = 0.0872
Absolute structure parameter	0(10)
Extinction coefficient	0.00038(10)
Largest diff. peak and hole	0.181 and -0.182 e.A^-3

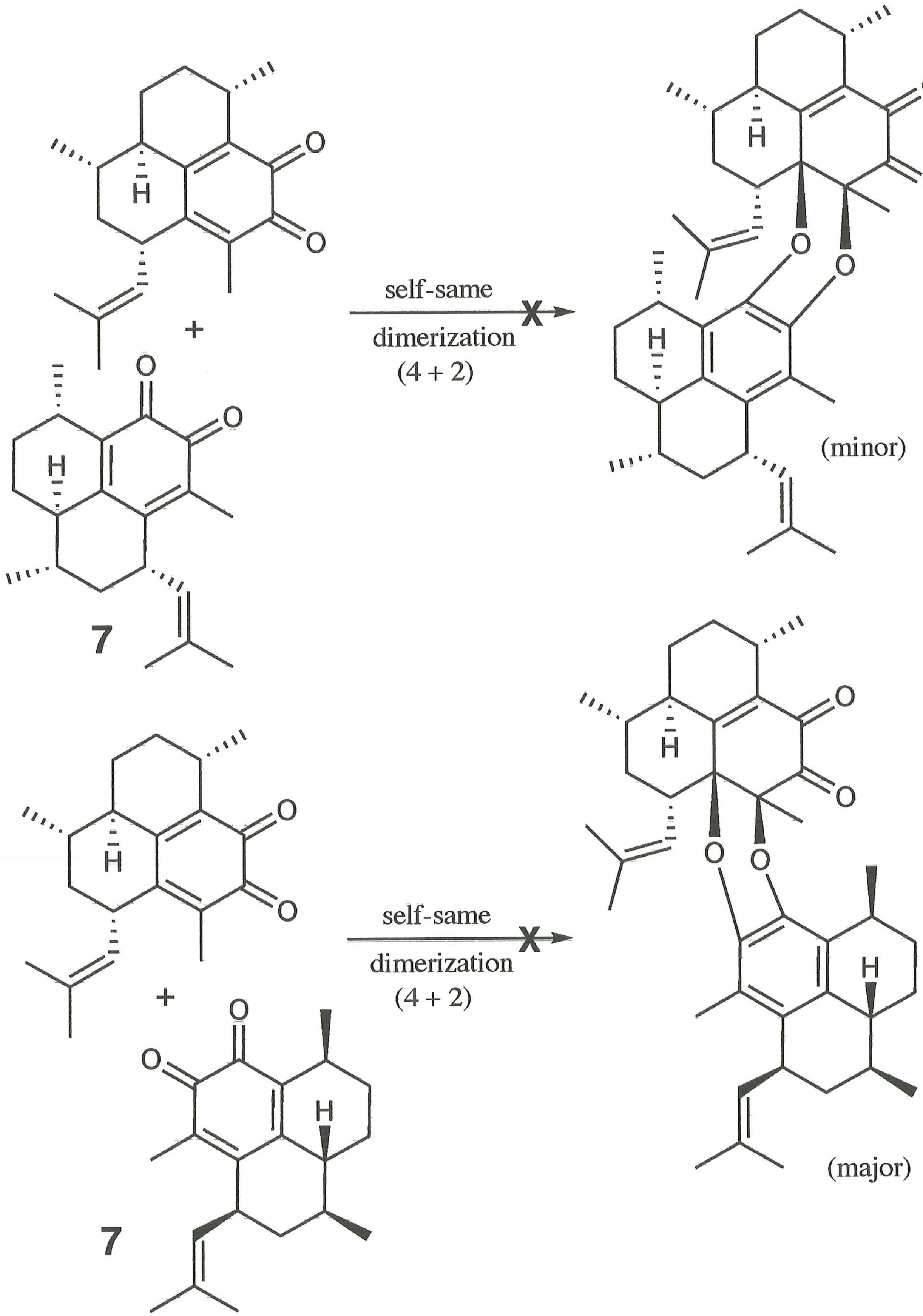

Figure S1. Predicted structures from self-same dimerization of *ortho*-benzoquinone **7** (not detected).

Figure S2. 1H NMR (500 MHz, CDCl3) spectrum of 1

Bisditerpene A

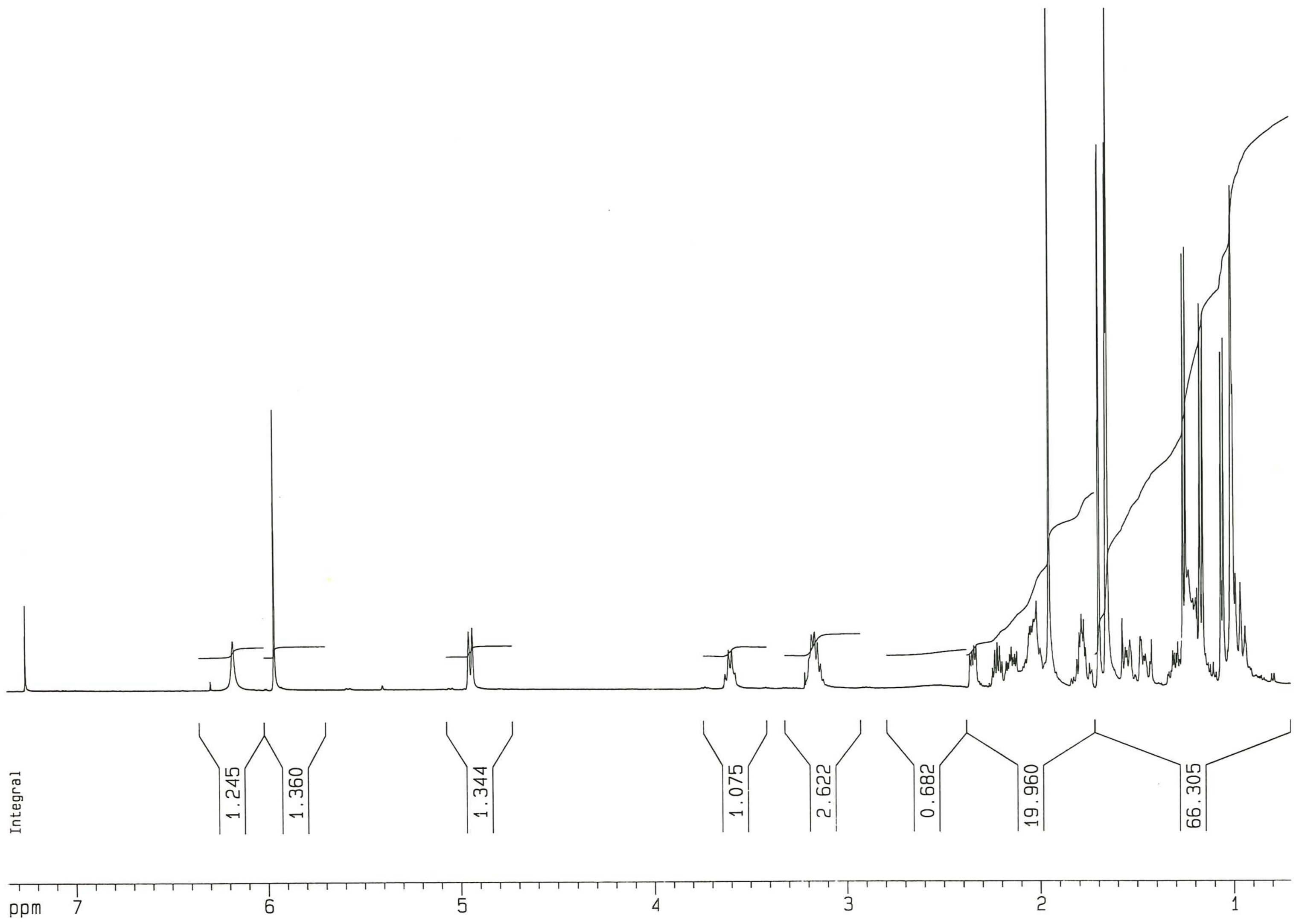

Current Data Parameters

NAME	pehdc10k2
EXPNO	1
PROCNO	1

F2 - Acquisition Parameters

Date_	20020710
Time	16.27
INSTRUM	spect
PROBHD	5 mm Multinu
PULPROG	zg30
TD	32768
SOLVENT	CDCl3
NS	32
DS	2
SWH	7246.377 Hz
FIDRES	0.221142 Hz
AQ	2.2610421 sec
RG	203.2
DW	69.000 usec
DE	6.00 usec
TE	300.0 K
D1	1.00000000 sec
P1	10.00 usec
SFO1	500.1328885 MHz
NUC1	1H
PL1	0.00 dB

F2 - Processing parameters

SI	16384
SF	500.1300135 MHz
WDW	EM
SSB	0
LB	0.30 Hz
GB	0
PC	1.00

1D NMR plot parameters

CX	20.00 cm
F1P	7.357 ppm
F1	3679.48 Hz
F2P	0.707 ppm
F2	353.72 Hz
PPMCM	0.33249 ppm/cm
HZCM	166.28813 Hz/cm

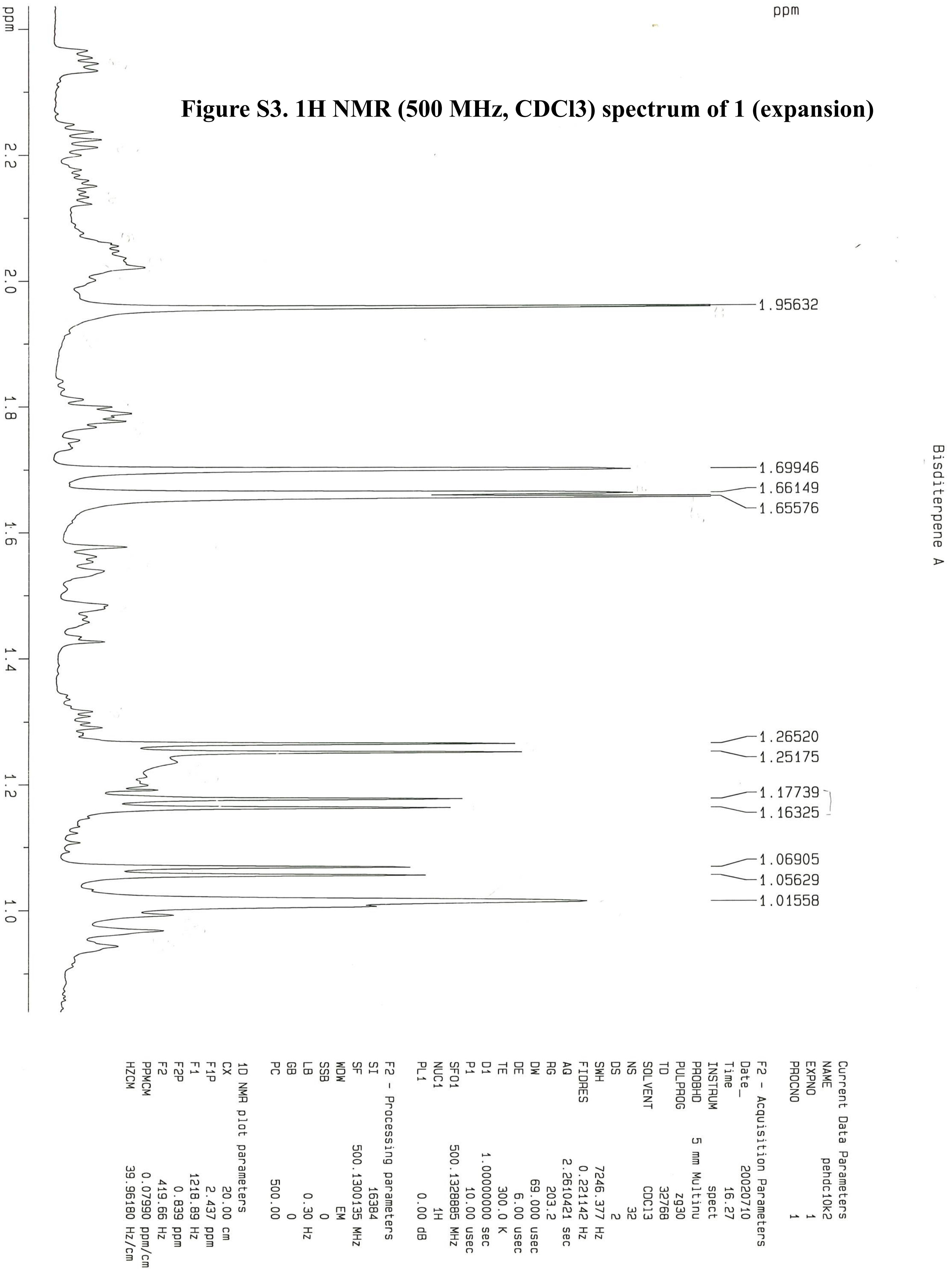

Figure S3. 1H NMR (500 MHz, CDCl3) spectrum of 1 (expansion)

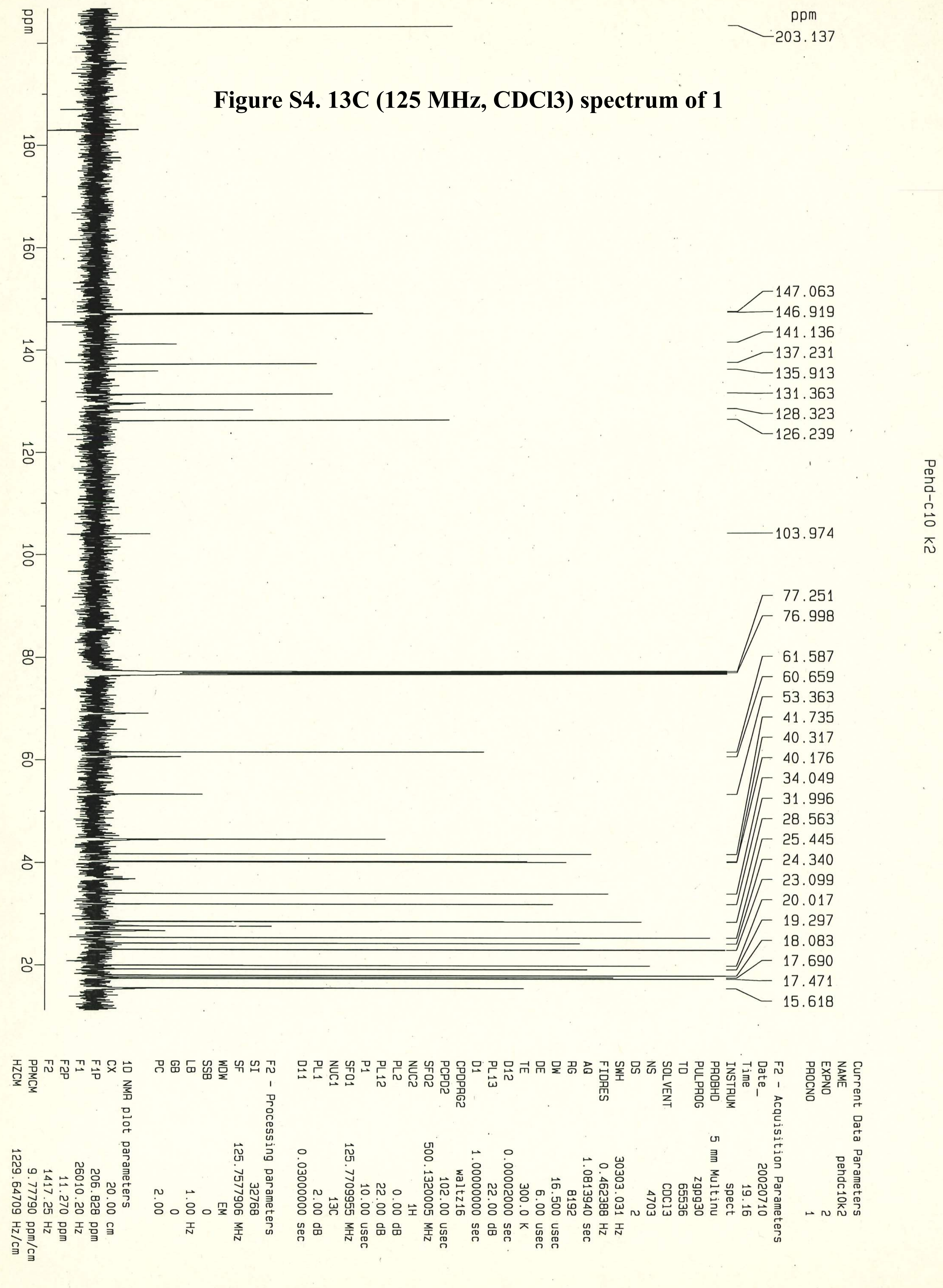

Figure S4. 13C (125 MHz, CDCl3) spectrum of 1

Figure S5. 13C (125 MHz, CDCl3) spectrum of 1 (expansion)

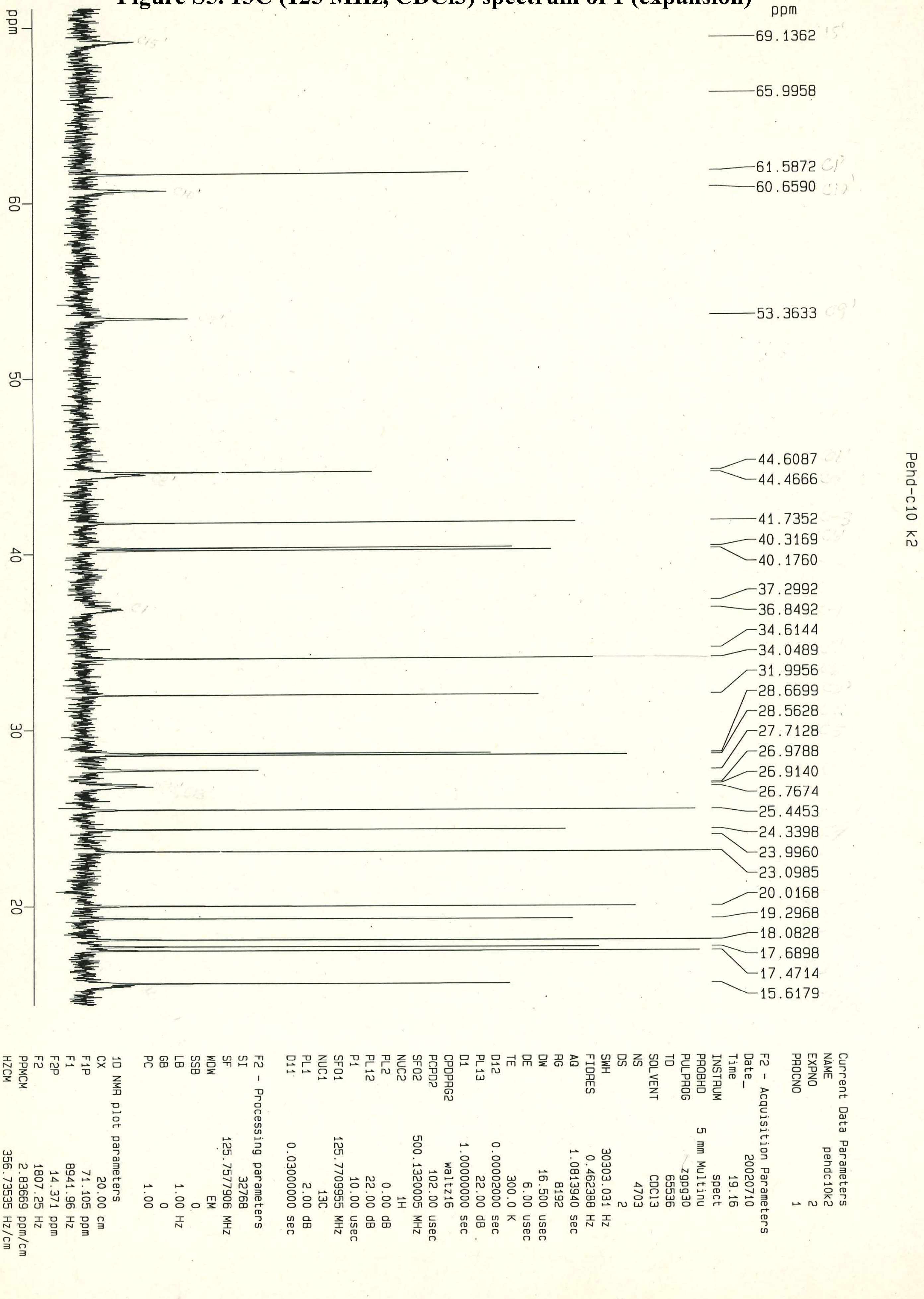

Figure S6. COSY spectrum of 1

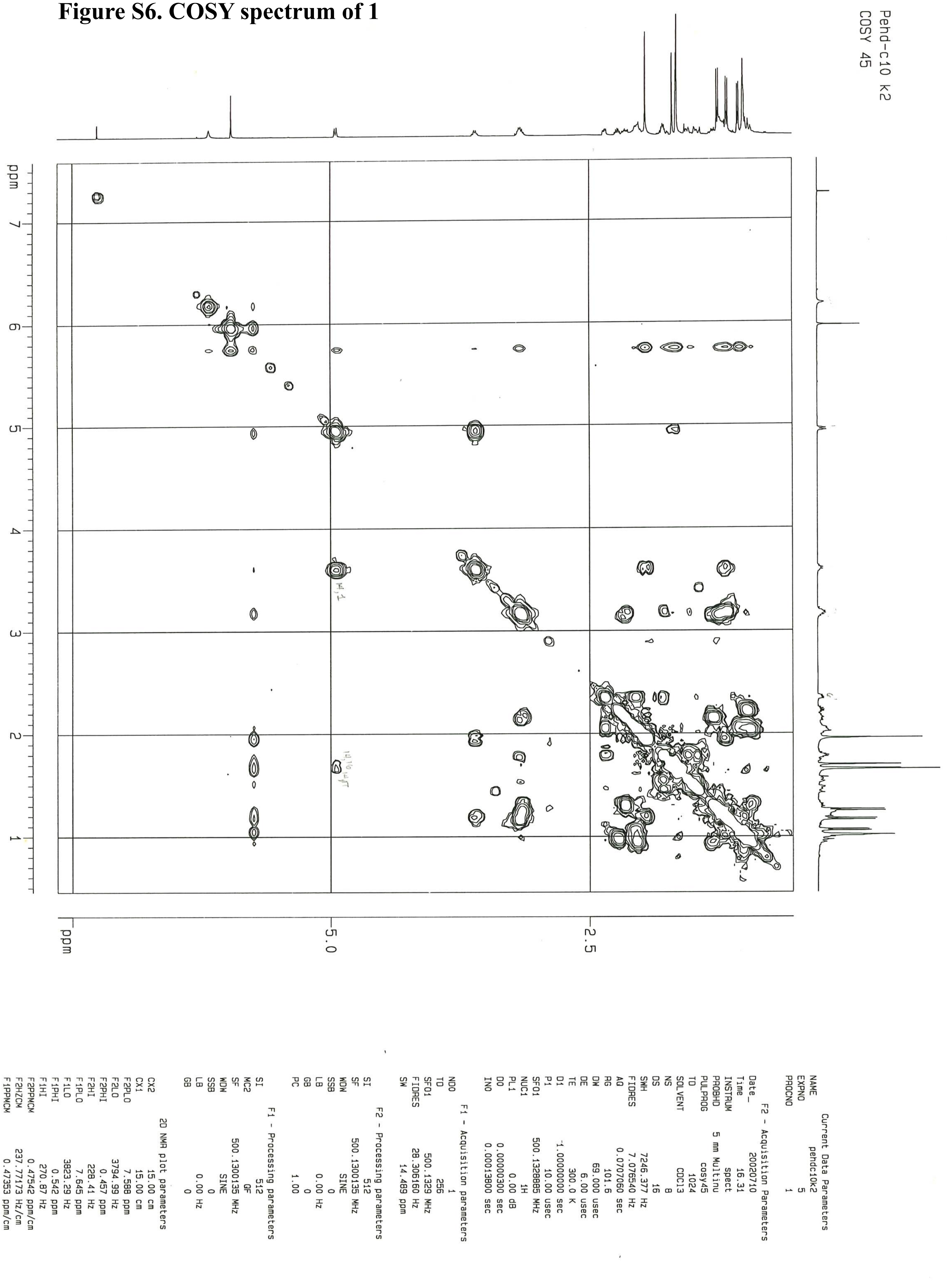

Figure S7. HMQC spectrum of 1

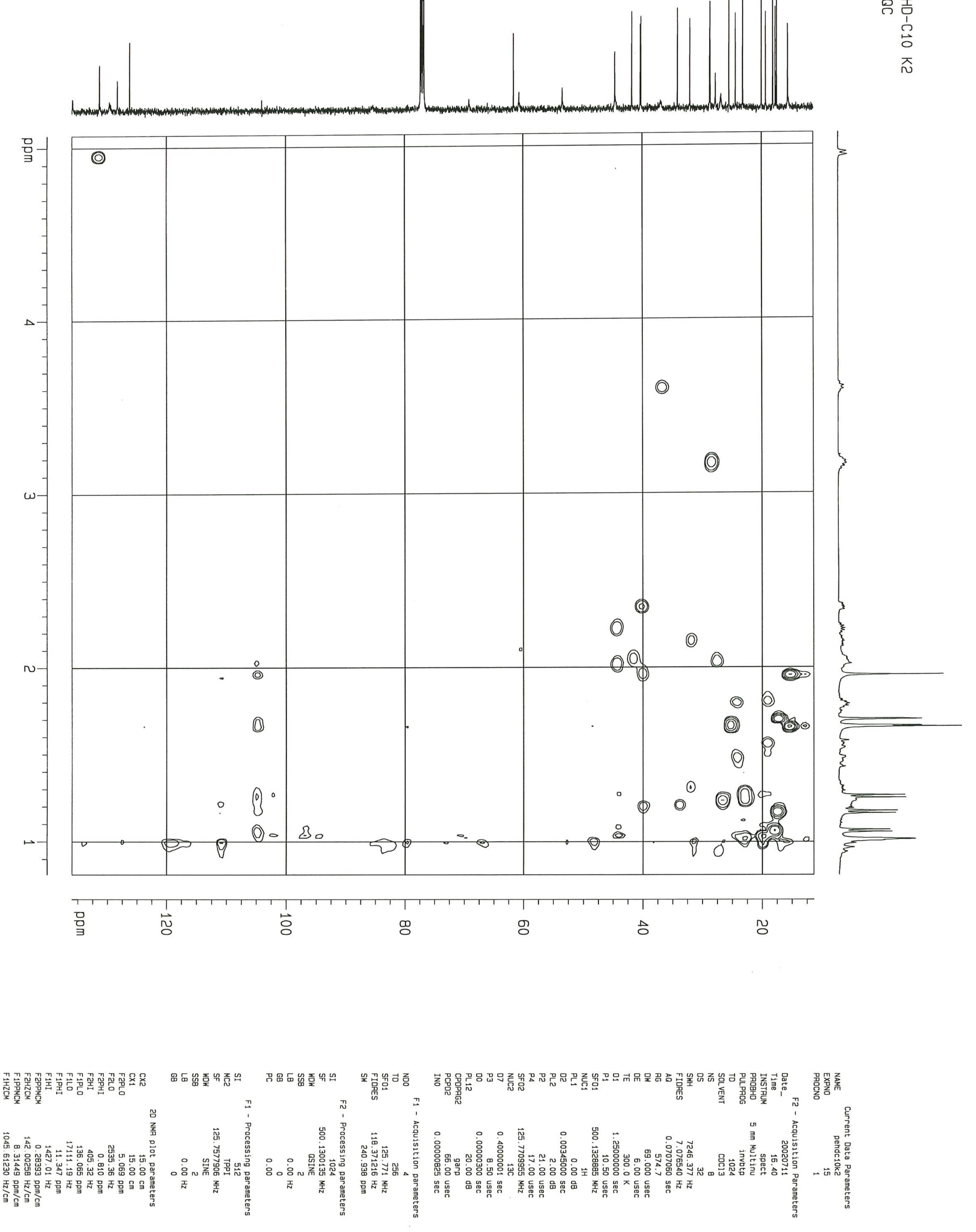

Figure S8. HMBC spectrum of 1

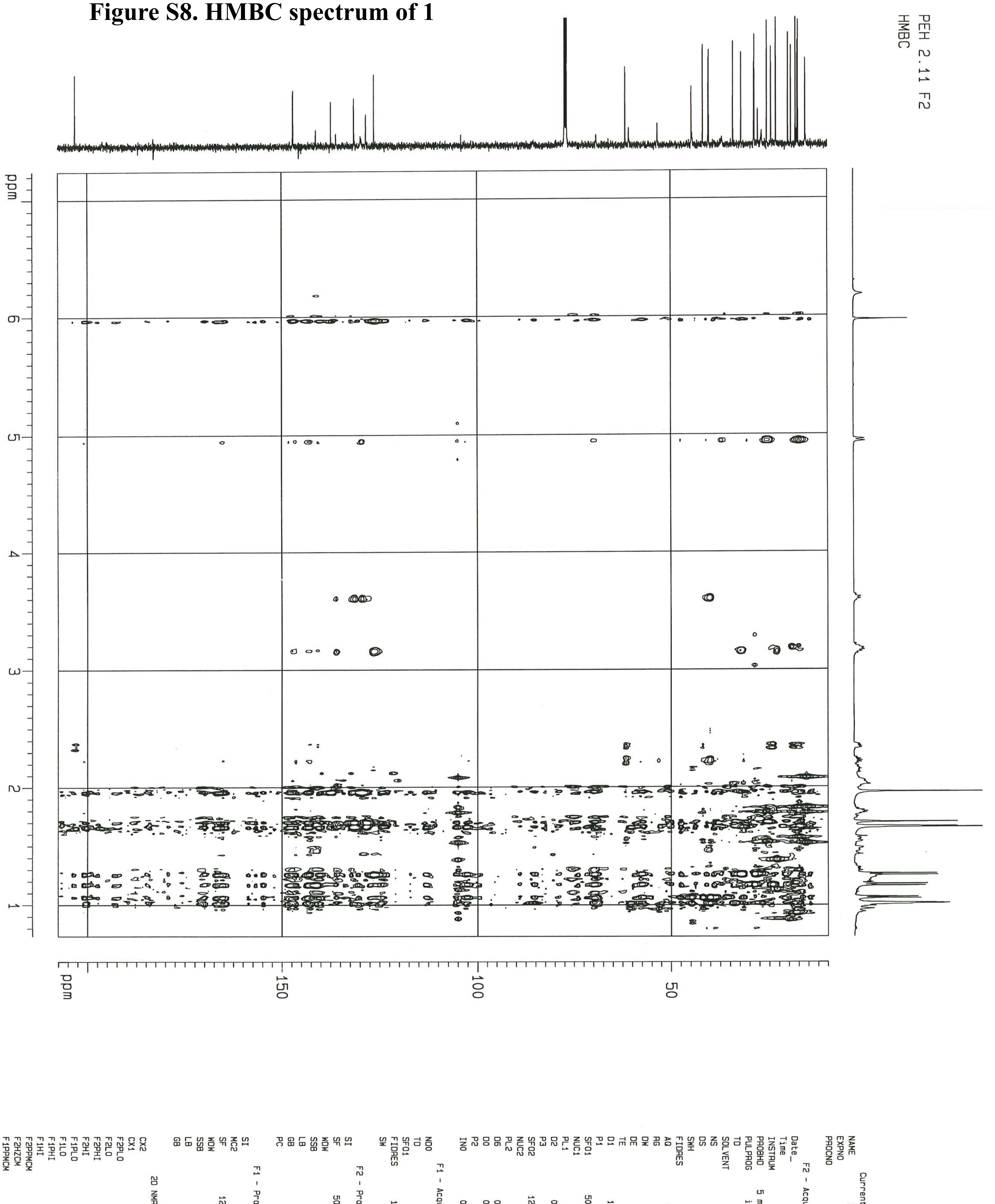

Figure S9. HMBC spectrum of 1 (expansion)

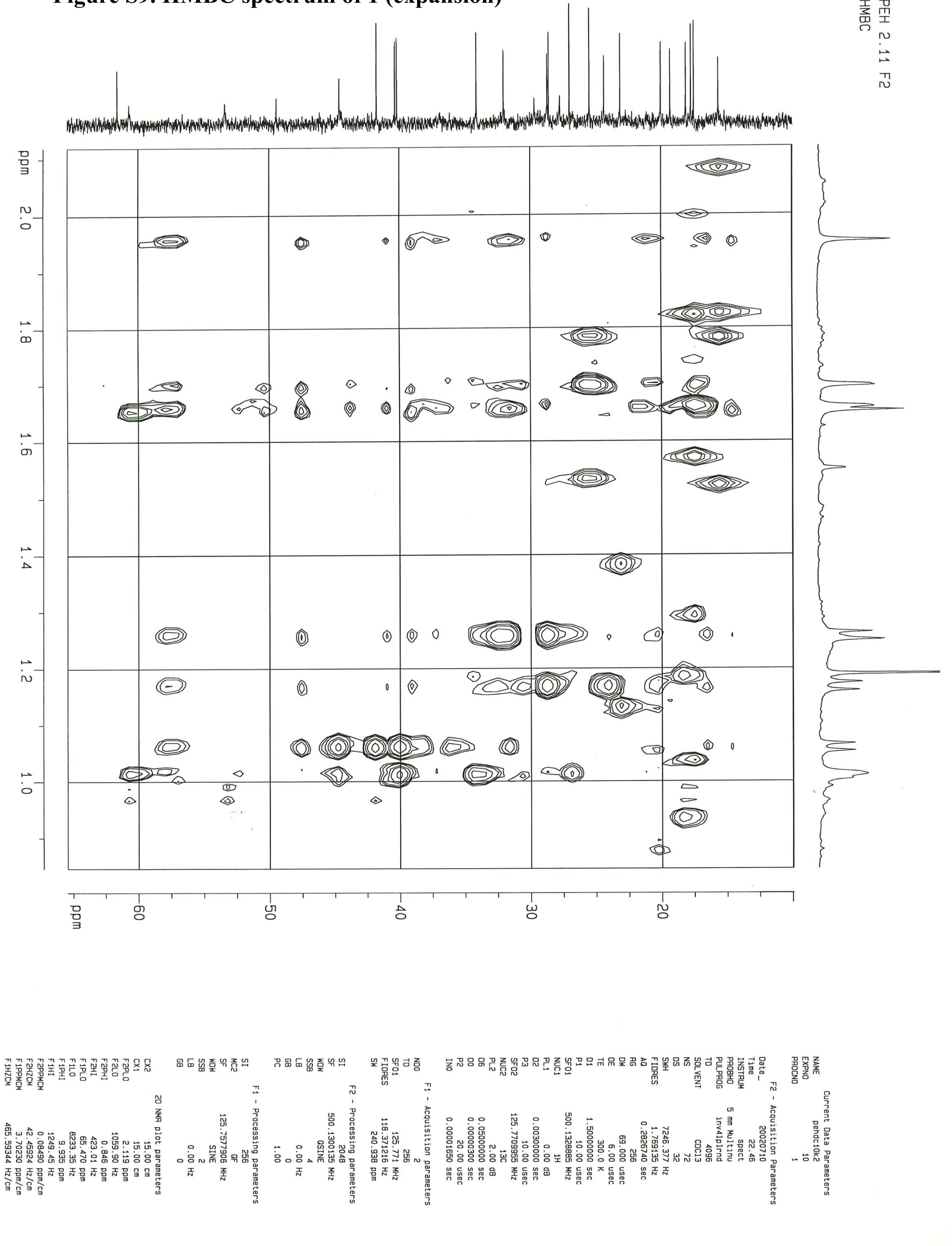

Figure S10. HMBC spectrum of 1 (expansion)

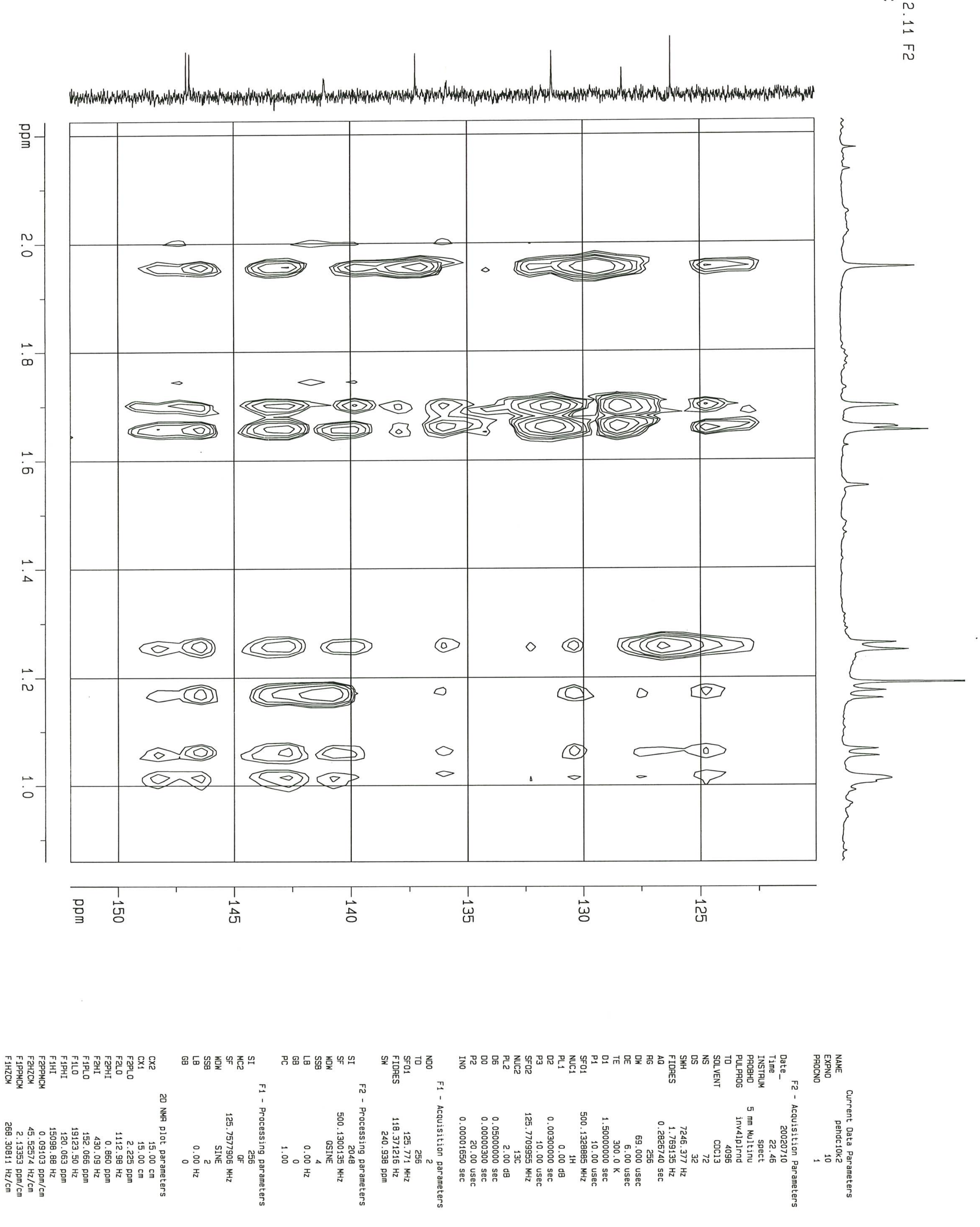

Figure S11. NOESY spectrum of 1

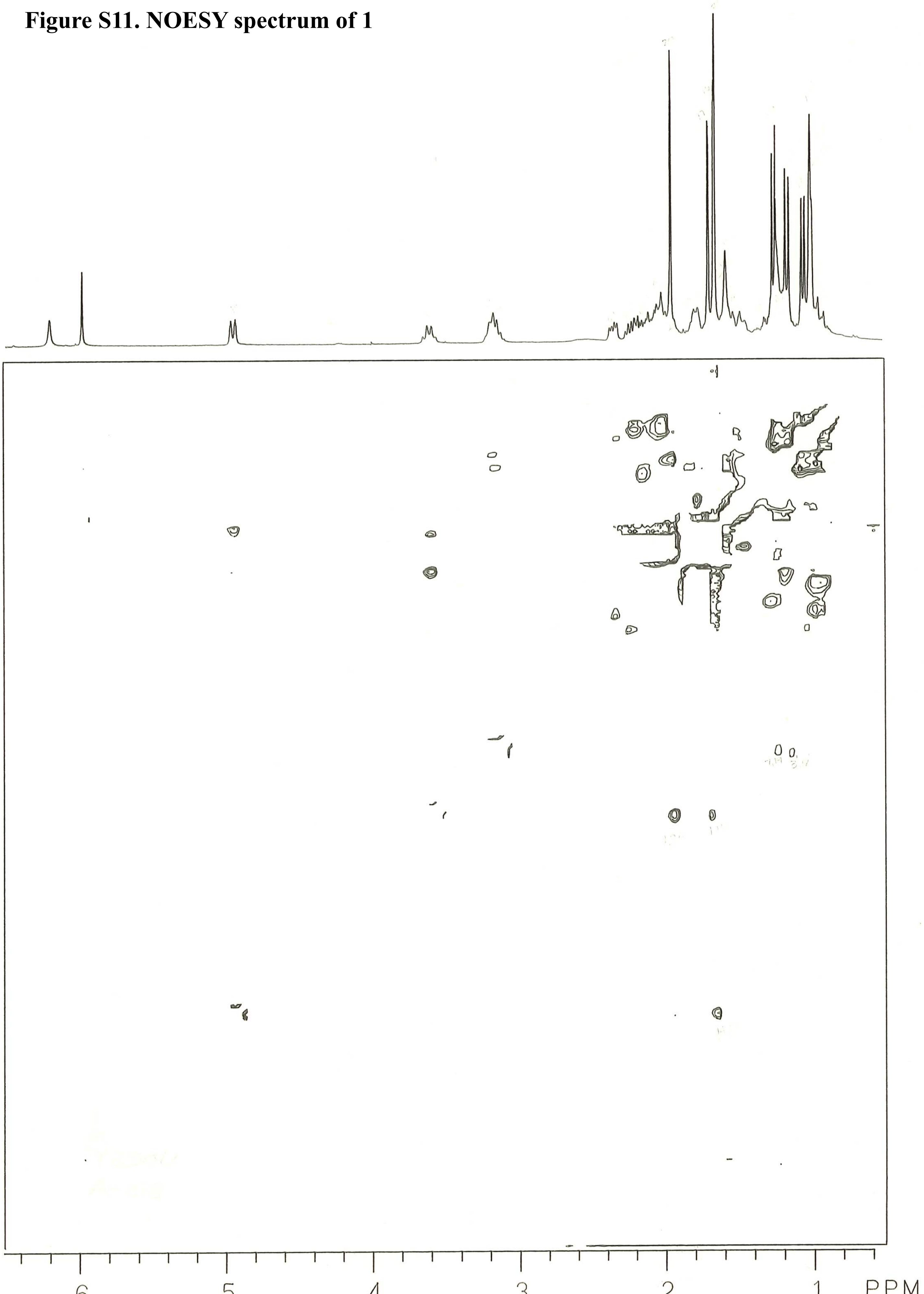

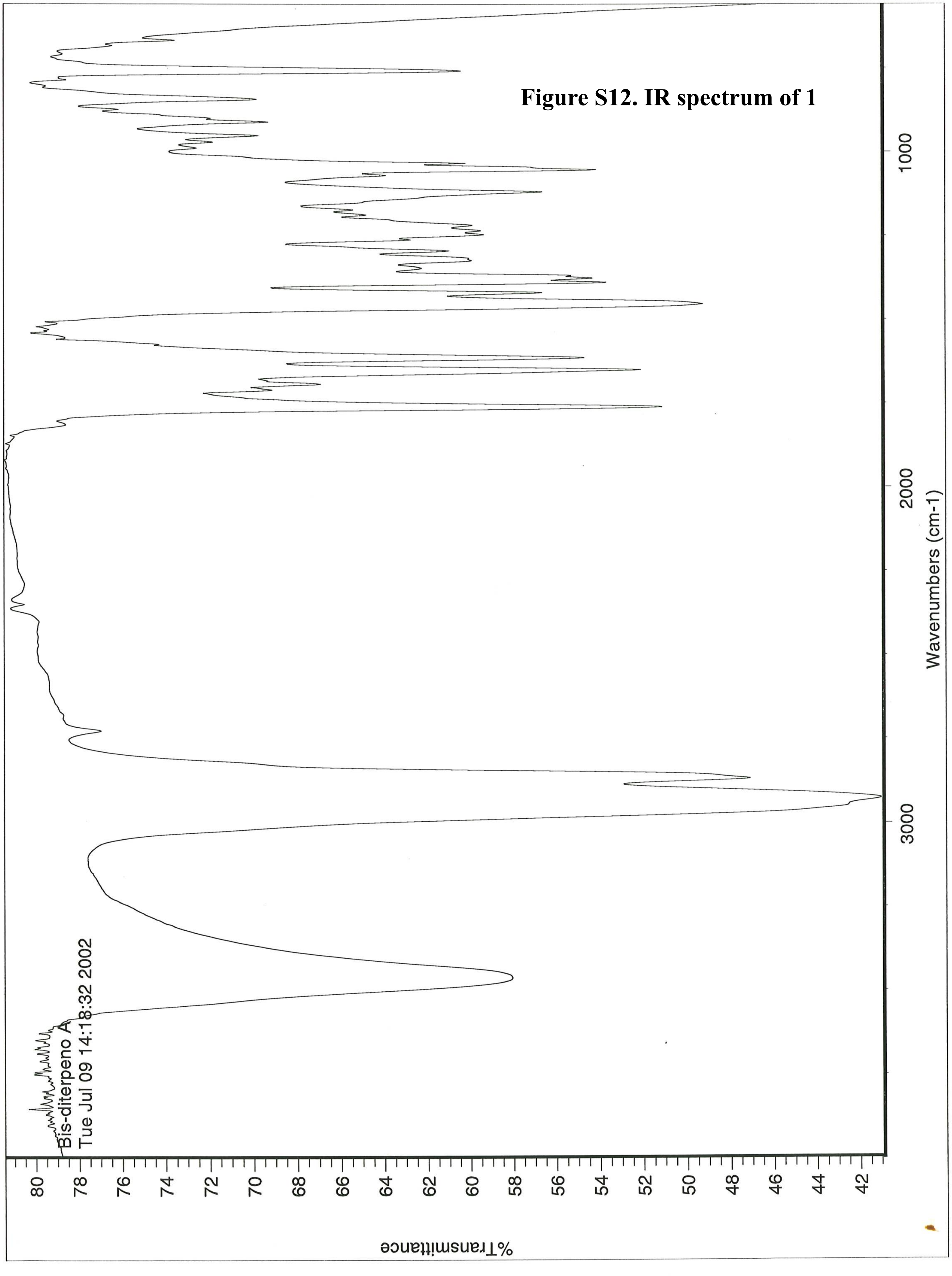

Figure S12. IR spectrum of 1

Figure S13. 1H NMR (500 MHz, CDCl3) spectrum of 2 (expansion)

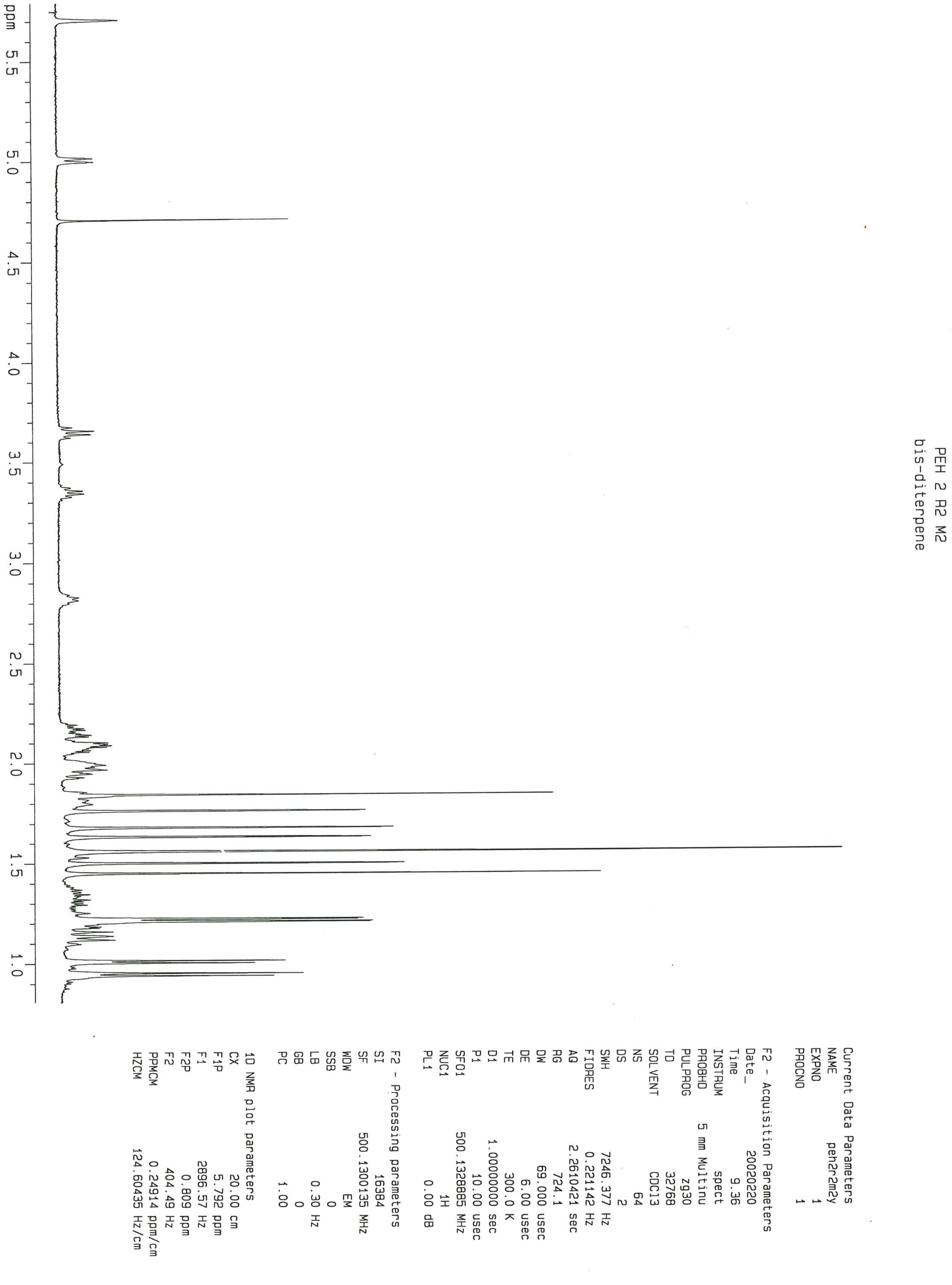

Figure S14. 1H NMR (500 MHz, CDCl3) spectrum of 2 (expansion)

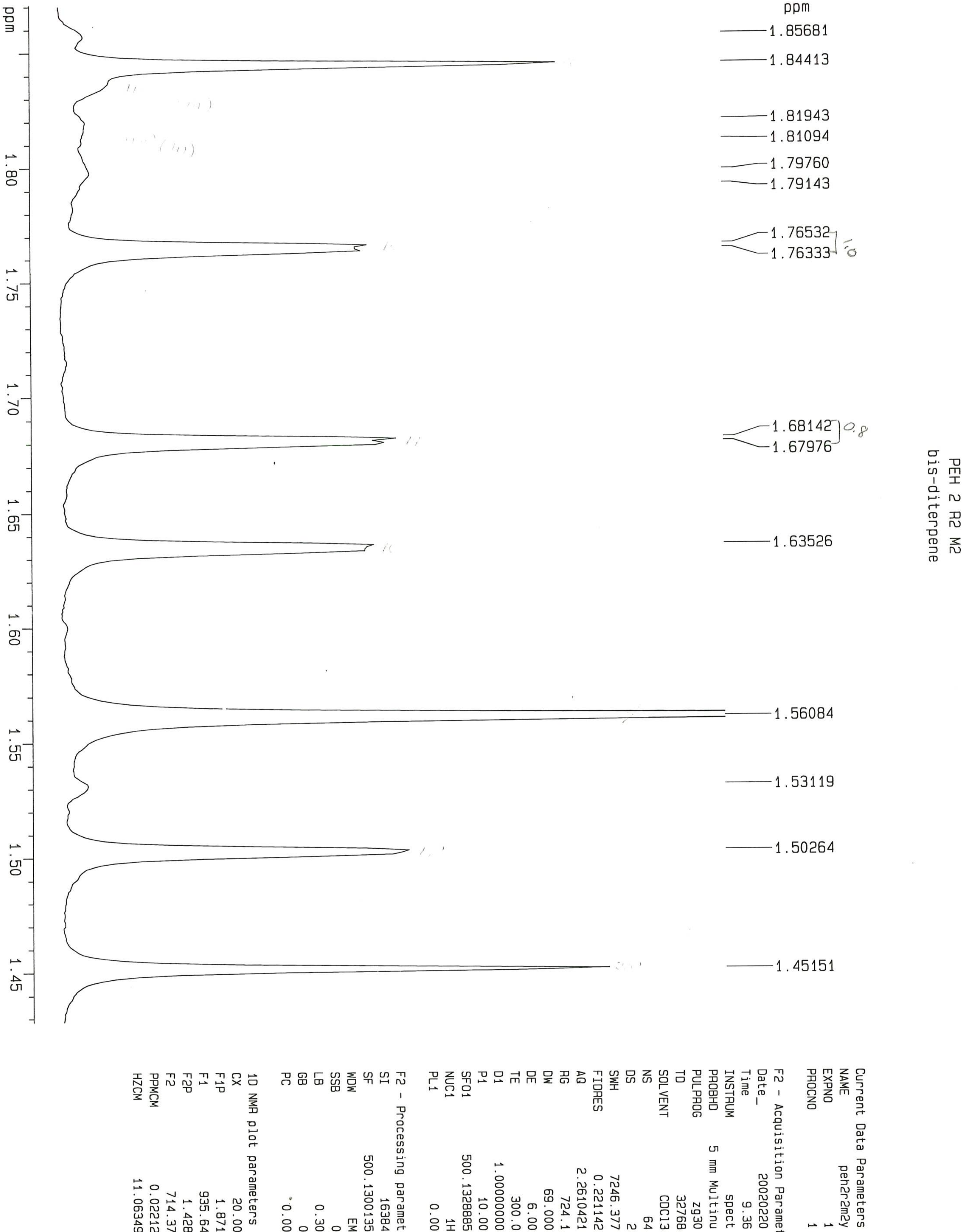

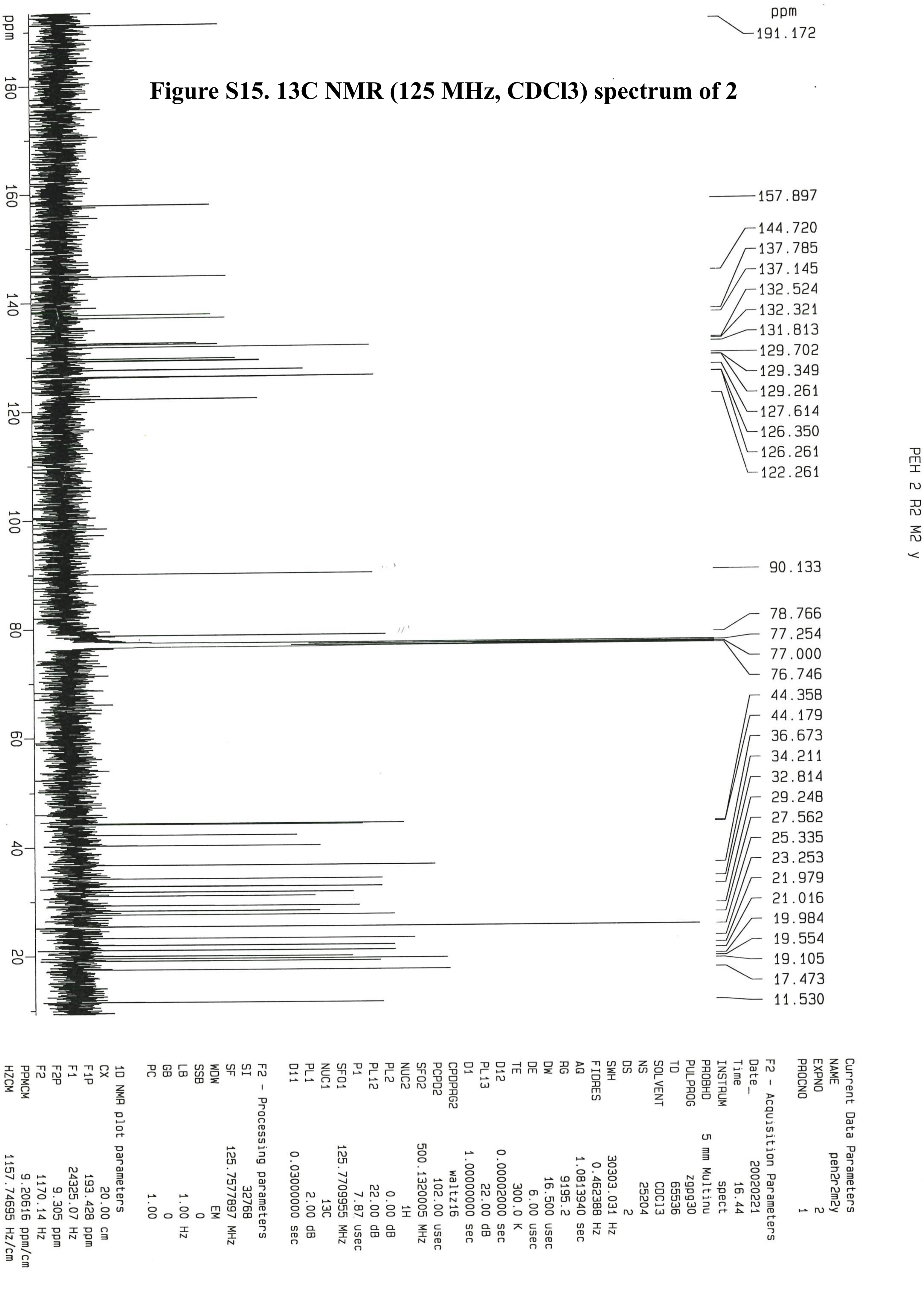

Figure S15. 13C NMR (125 MHz, CDCl3) spectrum of 2

Figure S16. 13C NMR (125 MHz, CDCl3) spectrum of 2 (expansion)

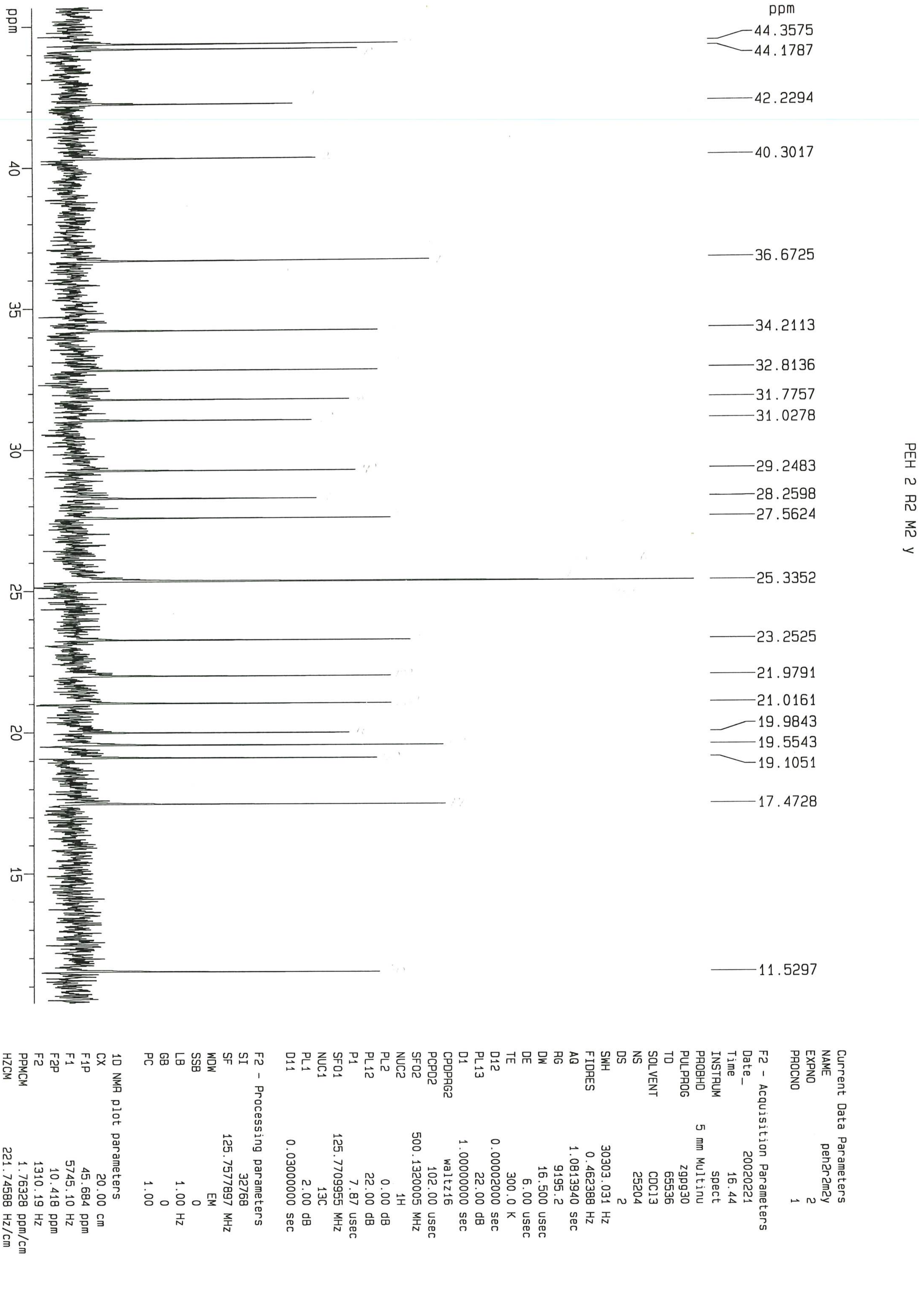

Figure S17. COSY spectrum of 2

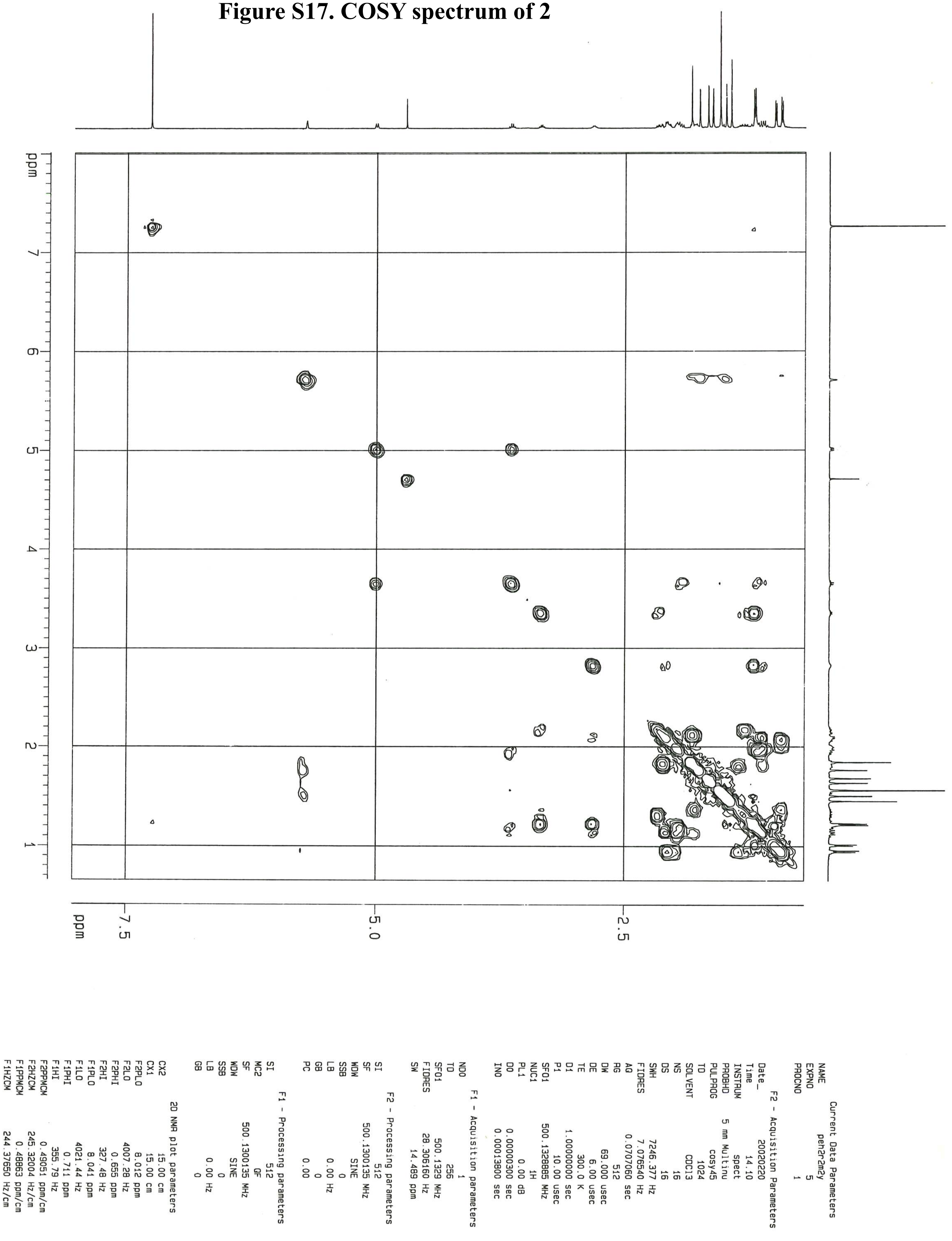

Figure S18. HMQC spectrum of 2

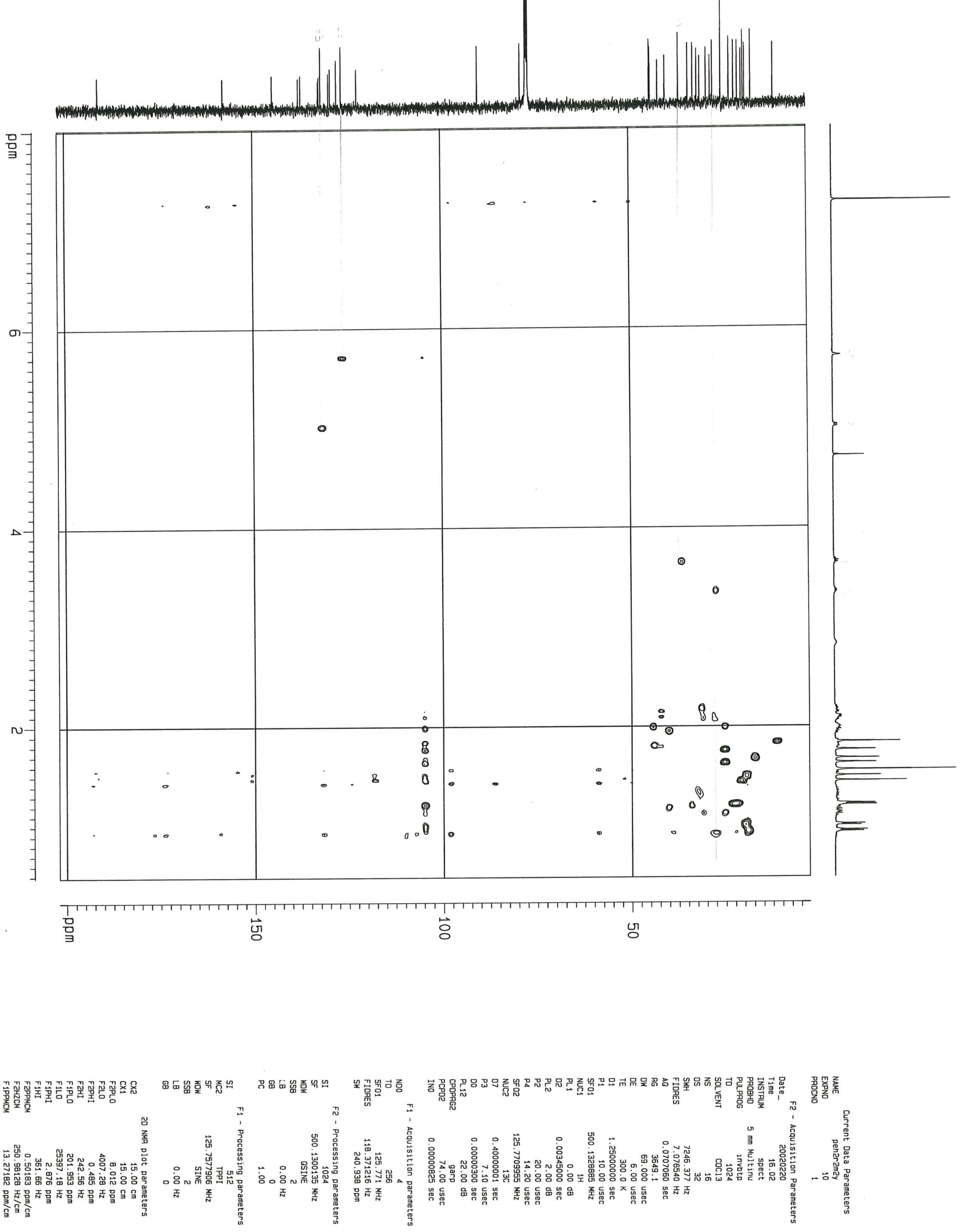

Figure S19. HMQC spectrum of 2 (expansion)

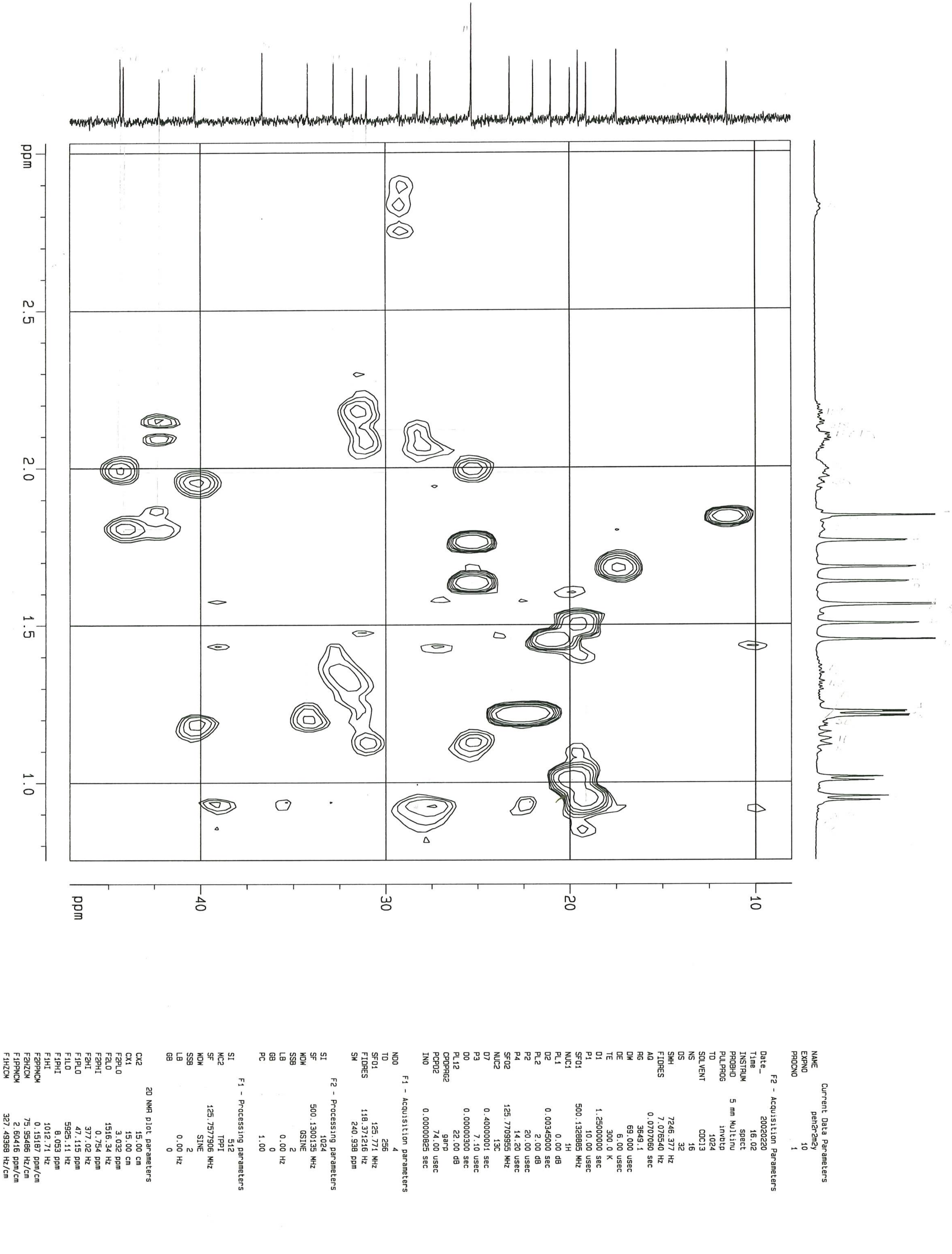

Figure S20. HMBC spectrum of 2

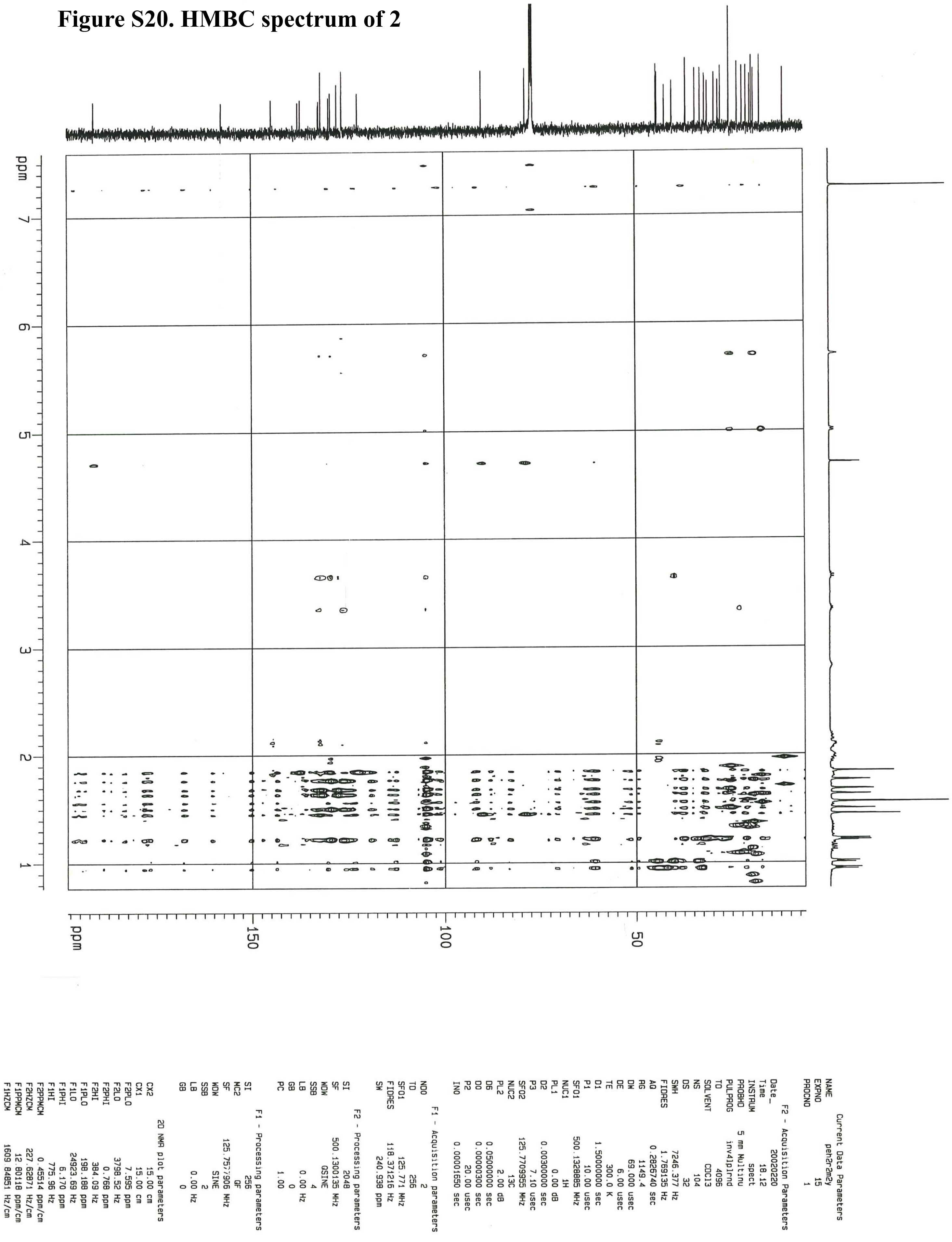

Figure S21. HMBC spectrum of 2 (expansion)

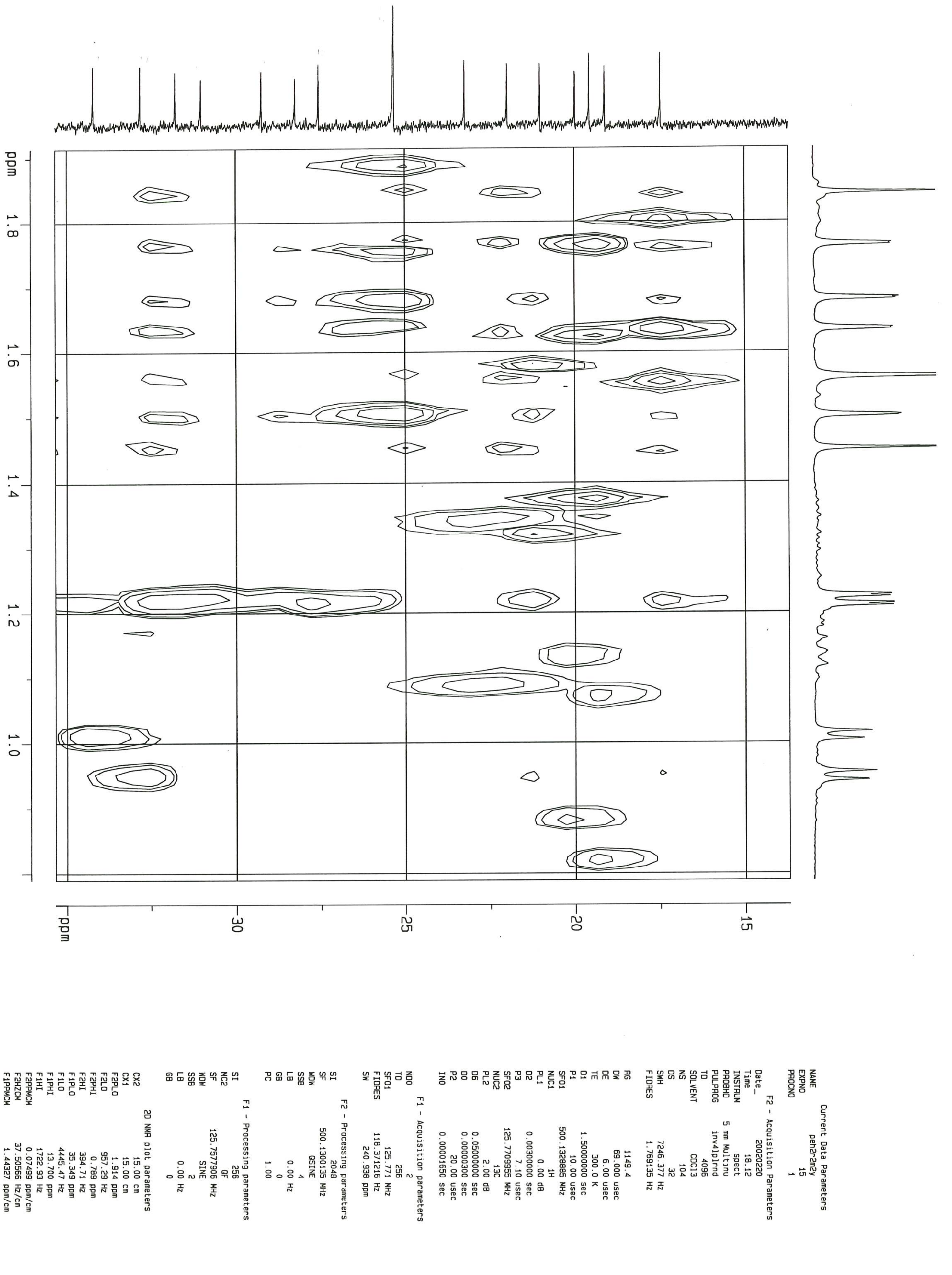

Figure S22. HMBC spectrum of 2 (expansion)

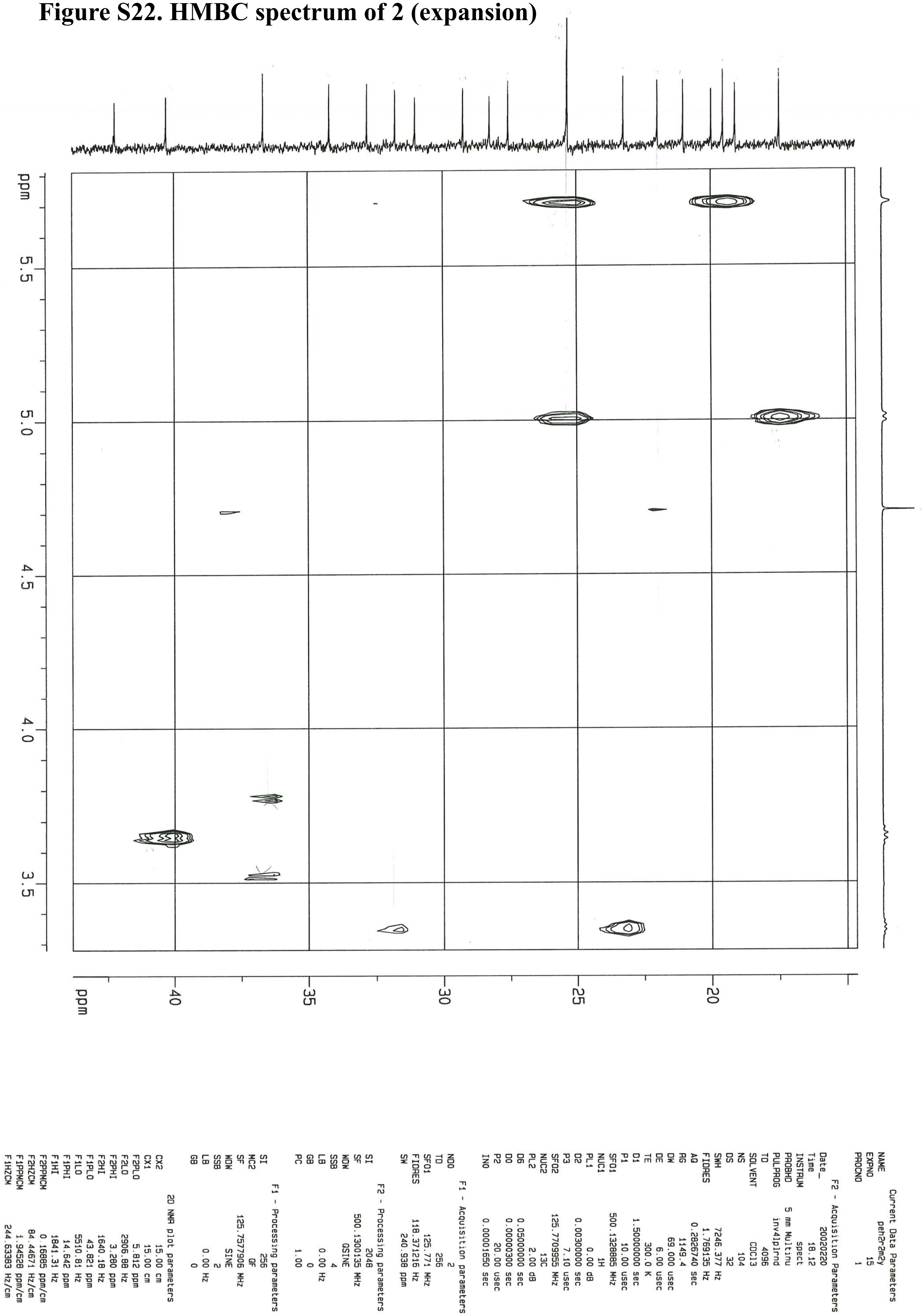

Figure S23. NOESY spectrum of 2

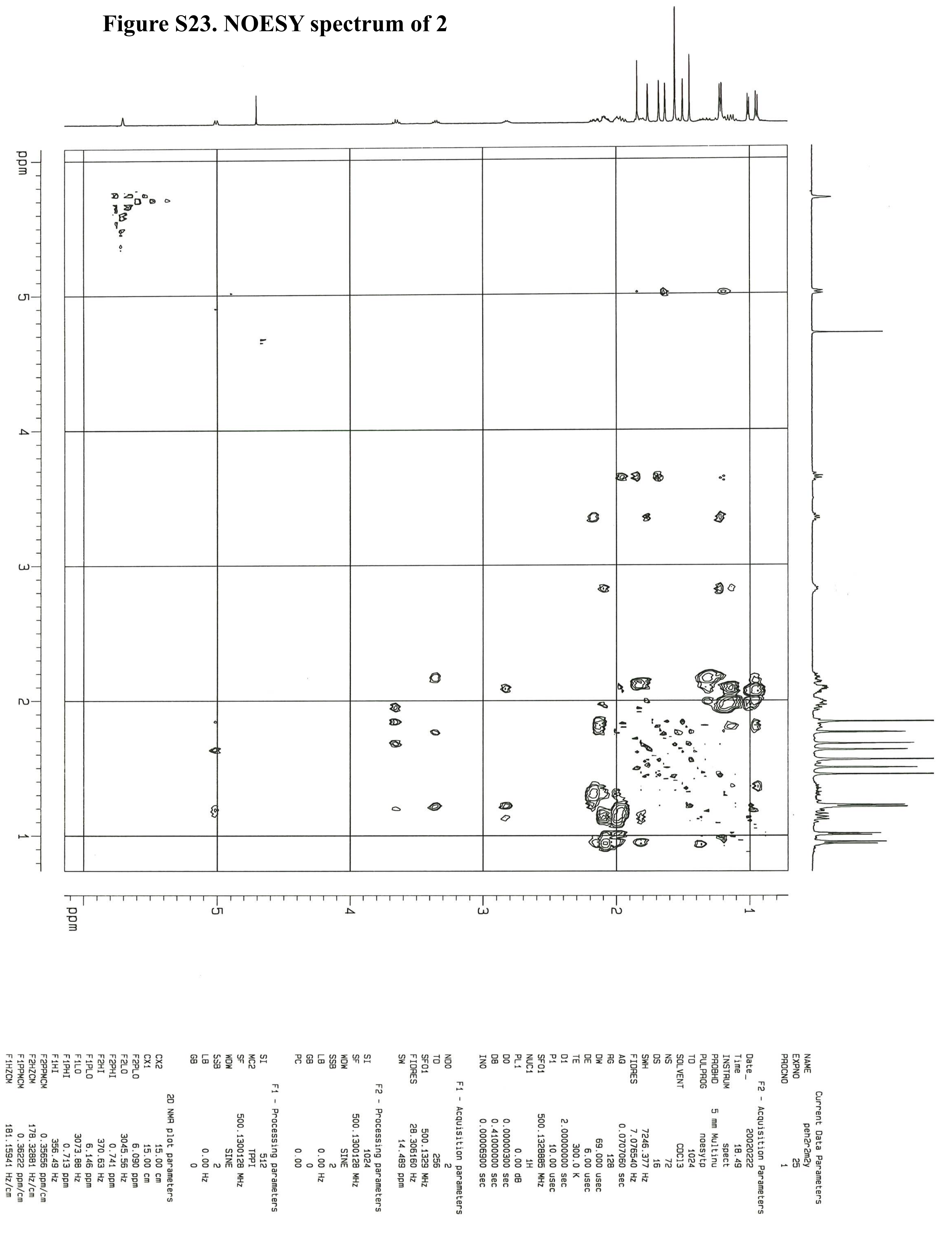

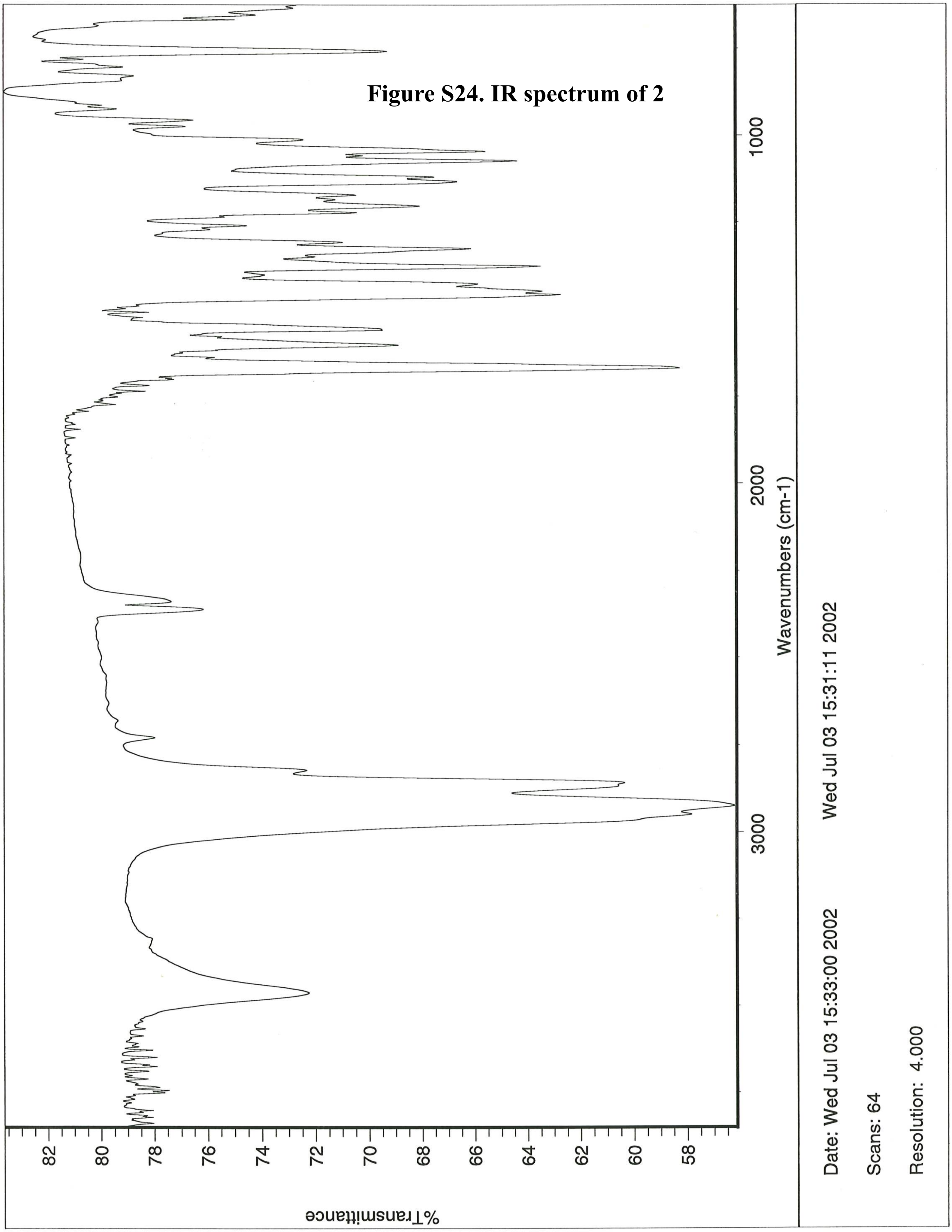

Figure S24. IR spectrum of 2
